# Gene expression profile of the skin in the 'hairpoor' (*Hr^Hp^*) mice by microarray analysis

**DOI:** 10.1186/1471-2164-11-640

**Published:** 2010-11-18

**Authors:** Bong-Kyu Kim, In-Cheol Baek, Hwa-Young Lee, Jeong-Ki Kim, Hae-Hiang Song, Sungjoo K Yoon

**Affiliations:** 1 Department of Biomedical Sciences, The Catholic University of Korea, 505 Banpo-dong, Seoul 137-701, Korea; 2 Division of Biostatistics and Department of Medical Life science, The Catholic University of Korea, 505 Banpo-dong, Seoul 137-701, Korea

## Abstract

**Background:**

The transcriptional cofactor, Hairless (HR), acts as one of the key regulators of hair follicle cycling; the loss of function mutations is the cause of the expression of the hairless phenotype in humans and mice. Recently, we reported a new *Hr *mutant mouse called 'Hairpoor' (*Hr^Hp^*). These mutants harbor a gain of the function mutation, T403A, in the *Hr *gene. This confers the overexpression of HR and *Hr^Hp ^*is an animal model of Marie Unna hereditary hypotrichosis in humans. In the present study, the expression profile of *Hr^Hp^/Hr^Hp ^*skin was investigated using microarray analysis to identify genes whose expression was affected by the overexpression of HR.

**Results:**

From 45,282 mouse probes, differential expressions in 43 (>2-fold), 306 (>1.5-fold), and 1861 genes (>1.2-fold) in skin from *Hr^Hp^/Hr^Hp ^*mice were discovered and compared with skin from wild-type mice. Among the 1861 genes with a > 1.2-fold increase in expression, further analysis showed that the expression of eight genes known to have a close relationship with hair follicle development, ascertained by conducting real-time PCR on skin RNA produced during hair follicle morphogenesis (P0-P14), indicated that four genes, *Wif1*, *Casp14*, *Krt71*, and *Sfrp1*, showed a consistent expression pattern with respect to HR overexpression in vivo.

**Conclusion:**

*Wif1 *and *Casp14 *were found to be upregulated, whereas *Krt71 *and *Sfrp1 *were downregulated in cells overexpressing HR in transient transfection experiments on keratinocytes, suggesting that HR may transcriptionally regulate these genes. Further studies are required to understand the mechanism of this regulation by the HR cofactor.

## Background

With a complex and dynamic structure, hair is generated by hair-producing follicles and has a patterned cycle of growth and remodeling, which consists of growth (anagen), regression (catagen), and rest (telogen) stages. There are many genes involved in mature hair follicle (HF) regulation [1].

One of these genes, *hairless *(*Hr*), is expressed in skin, specifically in the suprabasal cell layer of the interfollicular epidermis and in the lower portion of the HF epithelium; its expression is dependent on the hair cycle. *Hr *encodes a 130 kDa protein (HR), which contains a zinc finger domain and is localized in the nucleus [2], and acts as a transcriptional corepressor that regulates transcription through directly binding to the thyroid hormone receptor [3,4], vitamin D receptor [5], and retinoic acid-like orphan receptor α [6].

Various *Hr *mutant mice have been studied to understand the function of HR, and most *Hr *mutant mice are created by causing the loss of HR function in their cells, giving them a typical phenotype with a recessive inheritance mode [7-14]. Microarray analysis of the skin of *Hr^tm1Cct^/Hr^tm1Cct ^*mice has revealed that loss of HR function results in specific changes in the expression of epidermal differentiation-associated genes such as keratin10, loricrin, filaggrin, keratinocyte differentiation-associated protein (Kdap), and calmodulin 4, among other such mouse genes [7]. These results suggest that HR also plays a role in keratinocyte terminal differentiation through regulation of gene transcription.

The new *Hr *mutant mouse called 'hairpoor' (*Hr^Hp^*) that we reported recently has a phenotype that is inherited in an autosomal semidominant manner [15]. Therefore, the heterozygote shows poor hair distribution, whereas the homozygote displays total alopecia. The *Hr^Hp ^*mouse genome harbors a T-to-A substitution at position 403 in the noncoding exon 2 of *Hr*, and this mutation confers overexpression of HR in the mutant mouse skin [15]. This clearly distinguishes *Hr^Hp ^*mice from other *Hr *mutant mice with loss of function of *Hr*.

Marie Unna hereditary hypotrichosis (MUHH; OMIM-146550) is an autosomal dominant disorder that displays coarse and twisted hair development in early age in humans and progresses to alopecia as the patients grow older. Recently, the genetic cause of MUHH was found to be similar to the mutation found in *Hr^Hp^*, namely a mutation in the 5' UTR of the *HR *gene [16,17]. This makes the *Hr^Hp ^*mouse one of the valuable animal models for MUHH, and studying *Hr^Hp ^*mice will facilitate the understanding of MUHH pathogenesis. *Hr^N ^*mutant is another model for MUHH [15,18]. We recently reported that overexpression of HR is associated with alterations in the morphology and expression of a number of genes in the skin of the *Hr^Hp ^*mouse [16]. In the present study, we performed microarray analysis and compared *Hr^Hp^/Hr^Hp ^*skin with that of age-matched wild-type mice to identify the genes whose expression was affected by the overexpression of HR. We catalogued the genes showing differential expression in the mutant skin and found some of them to be tightly regulated by HR, which we confirmed using a reporter expression system.

## Results

### Hr overexpression preceded histological changes in the skin of *Hr*^*Hp*^/*Hr*^*Hp *^mice

Although we have shown that the overexpression of HR causes a number of morphological alterations in the skin, we know little about the molecular basis of these changes. Because the HR protein functions as a transcriptional co-repressor [4,5], we set out to identify the genes whose expression was specifically affected by HR overexpression using microarray analysis. To investigate the initial events underlying the morphological changes, we determined the time point at which morphological changes and *Hr *expression in the skin of *Hr^Hp^/Hr^Hp ^*mice first occurred.

**Figure 1:**
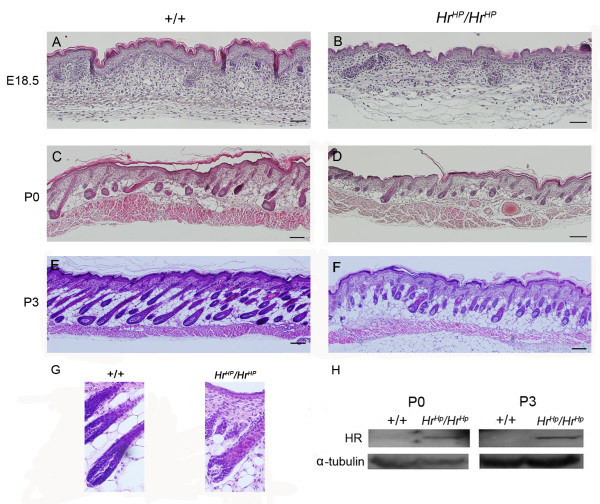
**HR was overexpressed without prominent morphological changes in the skin of *Hr^Hp^/Hr^Hp ^*and +/+ mice**. (A-F) Cross-sections of the skin of hairpoor mice (*Hr^Hp^*) during early stages (E18.5, P0, and P3). At E18.5 (A, B) and P0 (C, D), noticeable differences were not found between +/+ and *Hr^Hp^*/*Hr^Hp ^*skins. At P3 (E, F), There were shorter HFs detected in the *Hr^Hp^/Hr^Hp ^*skin. HF did not grow deep into the subcutis layer in *Hr^Hp^/Hr^Hp ^*skin than in the +/+. Scale bar = 100 μm. (G) Shape of hair bulbs in the +/+ and *Hr^Hp^*/*Hr^Hp ^*skins at P3. (H) HRoverexpression in the skin of *Hr^Hp^*/*Hr^Hp ^*mice at P0 and P3. Western blot analysis was performed with protein extracts from the backsides of mice at P0 and P3 using an HR antibody. The α-tubulin indicates equal amount of protein loading.

The fur of +/+ and *Hr^Hp^/Hr^Hp ^*mice showed noticeable differences by P7, so we examined the histology of the skin at the earlier time points by hematoxylin and eosin staining. At E18.5 and P0, we did not find any differences in the skins of +/+ and *Hr^Hp^/Hr^Hp ^*mice (Figure 1A-D) However we observed that HFs were much shorter and did not grow deep into the subcutis layer in *Hr^Hp^/Hr^Hp ^*skin than in the +/+ skin at P3. In addition, hair bulbs in the *Hr^Hp^/Hr^Hp ^*mice displayed short and round shape compared to the extended orval shape of the hair bulbs in the wiltype mice (Figure 1G). This observation clearly showed the morphological changes occurred in the mutant skin at P3.

Next, we compared HR protein expression in the skin of *Hr^Hp^/Hr^Hp ^*mice with that of +/+ mice by western blot analysis. The expression of HR was increased in *Hr^Hp^/Hr^Hp ^*skin compared with +/+ skin at P0 and P3, as shown in Figure 1H, indicating that HR was overexpressed in *Hr^Hp^/Hr^Hp ^*skin.

Based on these results, we decided to perform microarray analysis on the skin of mice at P0 as HR was overexpressed without prominent morphological changes in the skin of *Hr^Hp^/Hr^Hp ^*compared to that of +/+ mice at this stage.

### Microarray expression profile of genes differentially expressed in the skin of *Hr*^*Hp*^/*Hr*^*Hp *^mice

**Figure 2:**
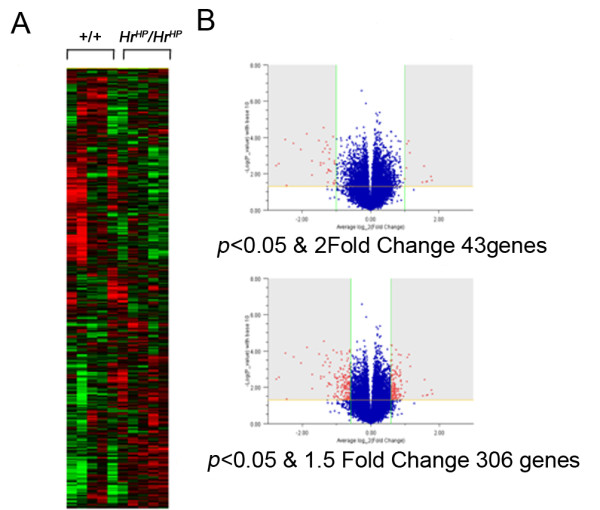
**Gene expression profile in *Hr^Hp^/Hr^Hp ^*skin using microarray**. (A) Hierarchical clustering represents differential expression of the genes between +/+ and *Hr^Hp^*/*Hr^Hp ^*skins. (B) Using DEG finding criteria, we found differential expression in 43 (>2-fold) and 306 genes (>1.5-fold) in the skin from *Hr^Hp^/Hr^Hp ^*mice compared with the skin from wild type mice.

Using 45,282 mouse probes, we performed microarray analysis and detected differential expression in 43 (>2-fold, *p *< 0.05), 306 (>1.5-fold, *p *< 0.05), and 1861 genes (>1.2-fold, *p *< 0.05) in the skin of *Hr^Hp^/Hr^Hp ^*mice compared with the skin of +/+ mice at P0 (Figure 2, Table 1), listed in Table 2. Among the 43 genes with > 2-fold expression, 33 were downregulated in *Hr^Hp^/Hr^Hp ^*skin at P0. The most strongly downregulated genes in the mutant skin included *Cidea *(0.14-fold), *Cyp2g1 *(0.18-fold) and *Krt71 *(0.3-fold). Contrasting this downregulation, the expression of 10 genes, *Sepina3h *(2.96-fold), *Hmgcs2 *(2.16-fold), and *Odc1 *(2.1-fold) in particular, were significantly upregulated despite HR being a transcriptional corepressor.

**Table 1:** Summary of microarray analysis result

Total number of probe	45282
Fold change ≥ 2	43
Up-regulated gene	10
Down-regulated gene	33
Fold change ≥ 1.5	306
Up-regulated gene	144
Down-regulated gene	162
Fold change ≥ 1.2	1,861
Up-regulated gene	1,001
Down-regulated gene	860

**Table 2:** Partial list of up-regulated genes and down-regulated genes in the skin of *Hr^Hp^/Hr^Hp ^*at P0 compared with that of age matched wild type (>1.5-fold, *p *< 0.05)

Target ID	Name	Fold change	*p*-value	*q*-value
3780021	Cidea	0.14795	0.003652023	0.042165577
2710593	Cyp2g1	0.17794	1.29E-04	0.023944824
2000592	Cyp2g1	0.20932	2.13E-04	0.023944824
7570053	BC018222	0.24346	4.54E-04	0.031620916
3060095	Krt71	0.30501	0.001969608	0.048669080
3360270	Cox7a1	0.30509	0.011595715	0.093860374
5080575	Sct	0.35145	1.42E-04	0.031326311
7380014	LOC384538	0.36474	0.002538937	0.042930078
1570546	Hbb-b1	0.36525	0.003431209	0.057411083
5090202	LOC381019	0.38211	6.05E-04	0.023944824
2810706	Fa2h	0.38835	0.002569014	0.042930078
2070669	Klk6	0.39619	4.11E-04	0.034431937
6560093	Sprr1a	0.40698	1.72E-04	0.031620916
3140327	2300006N05Rik	0.41588	0.049529381	0.150355126
1110079	2510042H12Rik	0.41670	0.009028142	0.064266519
1690201	Padi1	0.41805	2.69E04	0.031620916
3710154	Cox8b	0.42134	0.029399708	0.125326533
1300647	Mlana	0.42471	0.005797188	0.076856524
3850102	Epgn	0.43142	0.006935833	0.080685514
940338	S100a1	0.43397	7.18E-04	0.038738839
6770347	LOC208963	0.43461	0.001511473	0.038338360
7050255	Cryba4	0.43566	0.006667837	0.079783816
6900440	Eraf	0.44245	4.23E-04	0.036594329
4040435	Ndg2	0.44938	0.007298215	0.080852271
3800671	Scin	1.77886	0.037594639	0.116371433
6660292	Spo11	1.78048	0.002528848	0.034431937
3060255	Lamc2	1.78282	0.0230355	0.083502051
1660056	Ipas	1.78496	0.029101447	0.129397450
4290300	Sp5	1.79236	0.002254742	0.03833836
3140370	Nid1	1.82039	0.002087889	0.042930078
670328	Kb40	1.82237	0.024664344	0.084895074
1850687	Mef2a	1.82289	0.001537068	0.040725741
1850215	Zfp288	1.82622	4.35E-04	0.034981466
70408	Npn1	1.82836	0.029257525	0.091975622
6580113	Enpp1	1.82850	0.004107589	0.060941690
160066	Smad3	1.84147	1.70E-04	0.023944824
580360	Il31ra	1.84369	0.004864607	0.060941690
4850156	Cacna2d1	1.85595	0.011562648	0.068972844
630689	Cd109	1.86695	0.007151326	0.052147898
2070594	Dnajc6	1.86943	0.046158499	0.132801080
4260681	9830131B04Rik	1.93946	0.03095302	0.109141501
4760358	4933426E01Rik	2.00195	0.019683692	0.107044321
430008	Lrrn1	2.06697	0.004949943	0.037149792
6840121	Odc1	2.10213	2.28E-04	0.021604353
6280392	Hmgcs2	2.16618	1.57E-04	0.023944824
6290592	LOC226691	2.21928	7.06E-04	0.036594329
1450491	Serpina3h	2.96300	0.003770165	0.039381649

In addition, we also analyzed microarray data using *q*-value. We found differential expression of 23 (>2-fold, *q *< 0.05), 90 (>1.5-fold, *q *< 0.05), and 283 genes (>1.2-fold, *q *< 0.05) in the skin of *Hr^Hp^/Hr^Hp ^*mice compared with the skin of +/+ mice at P0. Fewer genes were found to be differentially expressed based on the *q-values *than on the *p-values*. While some of the genes with higher fold change in expression were found to be not differently expressed (*Cox7a1 *and *Hbb-b1*), many genes such as *Cidea*, *Cyp2g1, Krt71 Sepina3h*, *Hmgcs2 *and *Odc1 *showed significant differential expression with significant *q*-values (Table 2). All of 283 genes are listed in Additional file 1 and 2.

Using the database of Kyoto Encyclopedia of genes and genomes (KEGG), 306 genes (>1.5-fold, *p *< 0.05), which were identified to be affected by overexpression of HR in *Hr^Hp^/Hr^Hp ^*mice, were found to be involved in the biological pathways related to cell-cell signaling and communication, various cancers, metabolism, and regulation of the actin cytoskeleton (Table 3). These results suggested that pathways involved in the communication and proliferation of cells were affected by HR overexpression.

**Table 3:** KEGG pathway associated with *Hr *overexpression

KEGG Pathway	Gene Counts	*p*-value
Cell Communication	7	5.04E-11
Pancreatic cancer	5	5.36E-09
Regulation of actin cytoskeleton	4	2.70E-05
Prostate cancer	4	1.07E-06
Glioma	4	3.80E-07
Small cell lung cancer	4	1.54E-06
Melanoma	4	6.03E-07
Cytokine-cytokine receptor interaction	4	1.09E-04
Fatty acid metabolism	4	8.29E-08
Purine metabolism	4	1.37E-05
Focal adhesion	4	3.24E-05
MAPK signaling pathway	4	1.29E-04
Chronic myeloid leukemia	4	8.13E-07
Pantothenate and CoA biosynthesis	3	9.40E-07
PPAR signaling pathway	3	5.12E-05
Oxidative phosphorylation	3	1.23E-04
Colorectal cancer	3	6.81E-05
Bladder cancer	3	8.74E-06
Non-small cell lung cancer	3	8.07E-06

### Expression pattern of HF associated genes during HF morphogenesis

As the first step to delineating the function of the genes whose expression was affected by HR overexpression, we assessed genes directly associated with HF development. Because few genes were directly associated with HF morphogenesis and/or development in the 43 (>2-fold) or 306 (>1.5-fold) genes showing differential expression, we broadened our search to include the 1861 genes (>1.2-fold). Among those genes, we focused on the Wnt signaling pathway-associated genes (*Sfrp1*, *Wif1*, *Wnt7b*), caspase-14 (*Casp14*), Janus kinase-2 (*Jak2*), keratins (*Krt71*, *Krt15*) and fibroblast growth factor 10 (*Fgf10*) (Table 4). Wnt signaling is not only involved in HF development [19,20] but also in HF cycling [21]. HR is reported to play a role in these processes [15,21]. HF undergoes vast apoptosis during catagen; *Casp14 *[22] and *Jak2 *[23] play a role in apoptosis, and in addition, *Casp14 *is directly associated with epidermal cell differentiation [24]. *Krt71 *and *Krt15 *are some of the main constituents of structures that grow from the skin. *Krt71 *is expressed in the inner root sheath (IRS), specifically in Henle's and Huxley's layers [25], and *Krt15 *is expressed in the basal layer of the outer root sheath [26]. *Fgf10 *is expressed in dermal papilla, the outer root sheath, and keratinocytes [27].

**Table 4:** Validation of the differential expression of the selected genes in the mutant skin

		Homozygote/wild type Fold change	
		
Class	Gene name	Microarray	Real time PCR
Wnt signaling associated factor	*Sfrp1*	0.710	0.535
	*Wif1*	1.488	1.426
	*Wnt7b*	1.452	3.668
			
Apoptosis associated factor	*Casp14*	1.437	1.471
	*Jak2*	1.514	1.801
			
Keratin	*Krt15*	1.422	2.902
	*Krt71*	0.300	0.350
			
Growth factor	*Fgf10*	1.376	2.300

We validated the microarray analysis data using real-time PCR. This was carried out using gene-specific primers and the same RNA sources used for the microarray analysis. Results found by real-time PCR corroborated those from the microarray analysis, as shown in Table 4. Six upregulated genes, *Wif1*, *Wnt7b*, *Casp14*, *Jak2*, *Krt15*, and *Fgf10*, showed similar or higher upregulation by real-time PCR than microarray analysis. Two downregulated genes, *Sfrp1 *and *Krt71*, showed a similar fold reduction in expression with both measurement techniques.

To analyze the potential role of these genes in HF morphogenesis, we investigated their expression during early HF morphogenesis (P0-P14) by comparing their expression levels in the skin of mutant mice with those of wild-type mice. Further real-time PCR analysis revealed that the downregulated genes, *Krt71 *and *Sfrp1*, maintained their decreased expression status in the mutant skin throughout the various developmental stages. The relative expression levels of *Krt71 *and *Sfrp1 *mRNA in the mutant skin was decreased to 0.07-fold and 0.39-fold of those in the wild type skin at P14, respectively.

**Figure 3:**
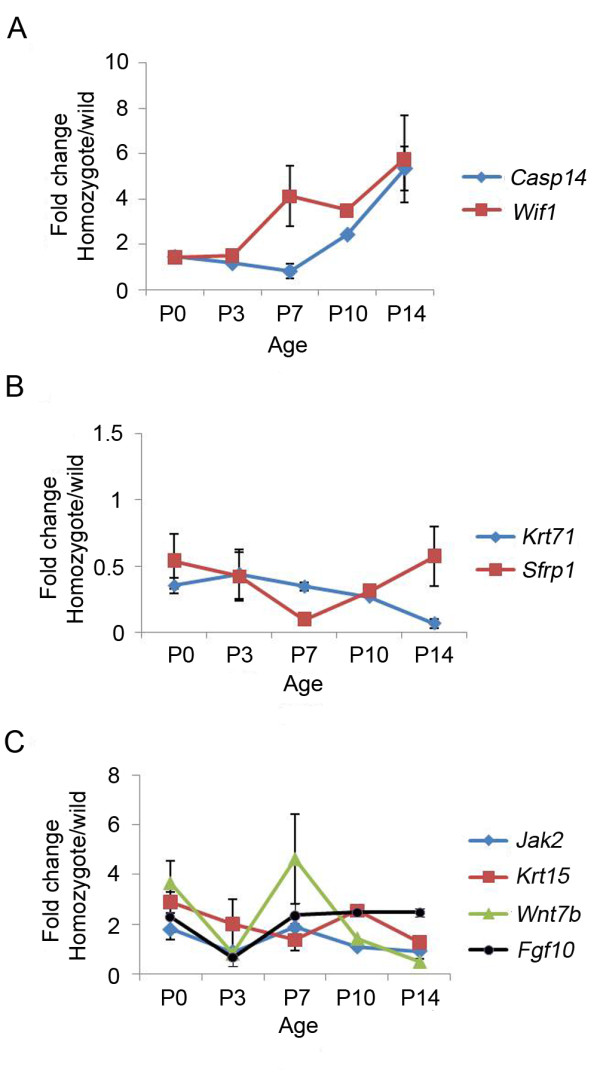
**Differential expression of the genes of interest during HF development with real-time PCR**. Four genes showed a consistent pattern of expression corresponding to *Hr*overexpression. *Wif1 *and *Casp14 *were upregulated (A) whereas *Sfrp1 *and *Krt71 *were downregulated (B) throughout the developmental stages. The remaining genes (*Wnt7b*, *Jak2*, *Krt15*, and *Fgf10*) displayed a complex expression pattern (C) during developmental stages (P0-P14). The Y-axis indicates the fold difference in expression, showing the relative expression level of each gene in *Hr^Hp^*/*Hr^Hp ^*mice compared with the skin of age-matched +/+ mice. The values are the average of the relative expression level of three mice, each measured in duplicate.

On the other hand, there were two subclasses of the upregulated genes; one group displayed a consistent expression pattern, whereas the other group showed an inconsistent pattern with respect to the HR expression. The relative expression levels of *Wnt7b*, *Krt15*, *Jak2*, and *Fgf10 *did not show a consistent pattern with respect to the HR expression in *Hr^Hp^/Hr^Hp ^*mice. In contrast, the levels of *Wif1 *and *Casp14 *mRNA gradually increased over time. Thus, by P14, the relative expression levels of *Wif1 *and *Casp14 *in *Hr^Hp^/Hr^Hp ^*skin were 5.77- and 5.35-fold higher than those of +/+ skin, respectively. This continuous increase in expression was consistent with the HR overexpression pattern in *Hr^Hp^/Hr^Hp ^*mice (Figure 3) [16].

### Expression of Wif1, Casp14, Sfrp1, and Krt71 in keratinocytes

**Figure 4:**
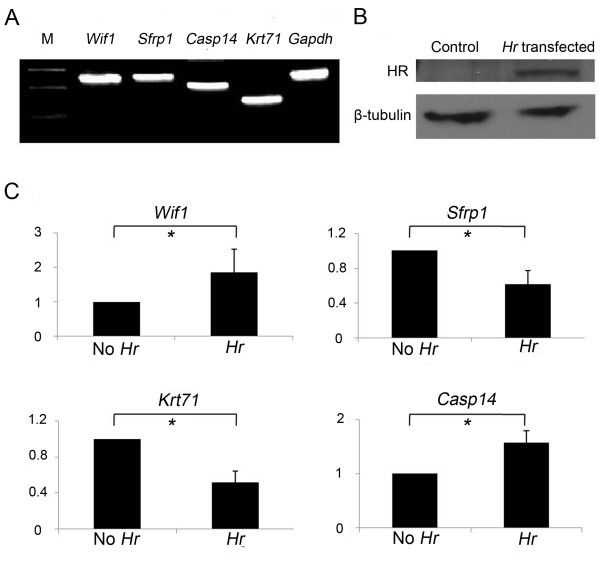
**Changes in expression of *Wif1*, *Sfrp1*, *Casp14*, and *Krt71 *in *Hr-*transfected PAM212 cells**. (A) Expression of *Wif1*, *Sfrp1*, *Casp14*, and *Krt71*in normal PAM212 cells using RT-PCR. M: size marker. (B) Western blot analysis showing the HR protein expressed in *Hr*-transfected PAM212 cells. β-tubulin indicates equal amount of protein loading. (C) Regulation of *Wif1*, *Sfrp1*, *Casp14*, and *Krt71 *gene expression by HR in *Hr*-transfected PAM212 cells by real-time PCR. The Y-axis indicates the fold difference in relative expression levels of each gene in HR-overexpressing cells compared with controls. *p value < 0.05. Results are the average of three independent experiments conducted in duplicate.

To further analyze whether the expressions of *Wif1*, *Sfrp1*, *Casp14*, and *Krt71 *were directly regulated by HR, we investigated changes in expression of these genes in the presence of overexpressed HR in a transient expression system using the mouse keratinocyte cell line, PAM212. RT-PCR revealed all the genes normally expressed in PAM212 cells (Figure 4A); transfection of PAM212 cells with *Hr *cDNA construct resulted in expression of HR (Figure 4B). The expression of all four genes was affected by the presence of HR: expression of *Wif1 *and *Casp14 *was increased 1.85- and 1.57-fold in HR-overexpressed cells compared to the mock-transfected PAM212 cells, respectively. In contrast, the relative expression of *Sfrp1 *and *Krt71 *was decreased to 0.61- and 0.52-fold (Figure 4C) in HR-expressing cells compared with control cells. These results were consistent with their expression pattern *in vivo *and strongly suggested that HR may directly regulate expression of these genes.

## Discussion

Recently, we reported the *Hr^Hp ^*mouse generated by *N*-ethyl-*N*-nitrosourea mutagenesis as an animal model of human MUHH [15]. As an initial step to delineate the molecular basis of the underlying mechanism for the *Hr^Hp ^*phenotype, we investigated the differential expression of genes in the skin immediately before morphological changes occurred in the *Hr^Hp^/Hr^Hp ^*mouse. Microarray analysis revealed that various biological pathways and the expression of many genes were affected by overexpression of HR.

Other studies have reported systematic screening of differentially expressed genes in the skin of mice with the *Hr *mutation, including analyses of gene expression in the skin of *Hr^N ^*mice, another *Hr*-overexpressing mutant, and *Hr^tm1Cct^*, an *Hr*-loss-of-function mutant [7,18]. *Hr^N ^*mutants harbor the mutation A402G, which abolishes the same uATG as in *Hr^Hp ^*mutants, indicating that they have an identical defect [16]. A comparison of our microarray analysis results with those reported for the *Hr^N ^*mutant did not show that the same genes displayed differential expression. This may be due to the difference in the developmental stage of the HF and epidermis (P0 vs. P7 or 5 weeks after birth) used in these analyses. However, many keratin-associated protein genes were detected in both mutants. At P0 in *Hr^Hp^/Hr^Hp ^*mice, the expression of *Krtap6-2*, *Krtap16-7*, and *Krtap16-3 *increased, whereas the expression of *Krtap5-1 *and *Krt71 *decreased. Similarly, *Krtap6-3*, *Krtap8-2*, *Krtap14*, *Krt1-1*, and *Krt1-3 *were downregulated in *Hr^N^*/*Hr^N ^*mice at P7 [18]. These results suggest that HR-regulated genes are associated with keratinocyte differentiation and/or hair-shaft structure. Furthermore, changes in the expression of keratin10, *Kdap*, and many epidermal differentiation-associated genes were also detected in *Hr^tm1Cct^/Hr^tm1Cct ^*mice at P12 [7]. Results suggest that mutation of *Hr *causes the abnormal expression of many keratin-associated genes during HF morphogenesis and result in disruption of normal hair formation [7].

Of four genes with consistent expression patterns with respect to HR overexpression, two genes, *Sfrp1 *and *Wif1*, belong to Wnt inhibitor families. Both inhibitors interfere with Wnt signaling transduction by binding directly to the WNT protein [28,29], but though they function in a similar fashion in the Wnt signaling pathway, they displayed completely different expression patterns in *Hr^Hp^/Hr^Hp ^*skin compared with age-matched +/+ skin. Surprisingly, *Sfrp1 *was downregulated by overexpression of the HR protein, whereas *Wif1 *was upregulated. Although we cannot rule out the possibility that this result may be caused by different locations of *Sfrp1 *and *Wif1 *expression in the skin, this difference is more likely to result from the differential transcriptional regulation of these genes, as seen in keratinocyte cells with HR overexpression (Figure 4). The *Hr *overexpression in keratinocyte cells results in suppression of *Sfrp1 *by 39% and activation of *Wif1 *by 85%, suggesting that these promoters respond to HR differently. It is not known whether HR functions as an activator for *Wif1 *transcription, and further study is required to understand its mechanism of action. Regulation of the Wnt pathway by HR through transcriptional regulation of *Wise*, *Soggy *and *Sfrp2 *is reported to be important for proper HF cycling. While expression of *Wise *and *Soggy *mRNAs were upregulated in the *Hr^tm1Cct^/Hr^tm1Cct ^*skin. Their expression levels were reduced in the skin of HR over-expressing transgenic mouse (2-fold). *Sfrp2 *was also shown to be downregulated in the *Hr^Hp^/Hr^Hp ^*skin. [16,21,30]. We may include two more Wnt inhibitors, *Sfrp1 *and *Wif1*, for being regulated by HR based on this work. Further study is required to understand their function(s) in HF development and/or cycling.

*Casp14 *is another gene upregulated by overexpression of HR, and is expressed in IRS and corneous cells of the outer root sheath in HFs [31]. It plays a role in the formation of the stratum corneum and terminal differentiation of keratinocytes [22,31]. The higher levels of *Casp14*mRNA suggest that an increase in terminal differentiation of keratinocytes occurs in the mutant skin. This result is comparable with our observation that the epidermis of the mutant skin shows increased differentiation in three-week-old mutant mice compared with age-matched wild-type mice [16]. Interestingly, *Casp14 *is also highly expressed in *Hr^tm1Cct^/Hr^tm1Cct ^*mice, which display the hairless phenotype and show an increase in terminal differentiation of the epidermis with loss of function of *Hr *[7]. Furthermore its striking up-regualtion is age specific, which closely resembles its expression pattern in the *Hr^Hp^/Hr^Hp ^*mice. Thus, despite having the opposite molecular defect, *Hr^Hp^/Hr^Hp ^*and *Hr^tm1Cct^/Hr^tm1Cct ^*mice exhibit increased expression of *Casp14*and displayed a similar increase in the terminal differentiation of keratinocytes, suggesting that, *Casp14 *may play an important role in proper differentiation of ketatinocyte. Thus, although it is not clear how HR regulates *Casp14*expression, both loss of *Hr *expression and *Hr *overexpression lead to alopecia through expression of *Casp14 *expression. *Krt71 *is also expressed in the IRS, specifically in Henle's and Huxley's layers; gene knockout mice show irregularly formed Henle's and Huxley's layers compared with the wild-type mice, indicating that *Krt71 *plays an important role in the formation of linear IRS intermediate filaments [25]. Results show that the downregulation of *Krt71*expression in the skin of *Hr^Hp^/Hr^Hp ^*mice is comparable with that in *Krt71 *knockout mice in that IRSs in *Hr^Hp^/Hr^Hp ^*mice were also abnormal.

## Conclusion

*Wif1 *and *Casp14 *were found to be upregulated, whereas *Krt71 *and *Sfrp1 *were downregulated by overexpression of HR. These results suggest that HR may transcriptionally regulate these genes. Clearly, further studies are required to delineate the molecular mechanisms underlying how HR regulates its target genes and to elucidate the role of *Hr *in HF development, leading to a better understanding of MUHH.

## Methods

### Histological study of the skins of wild-type and *Hr*^*Hp*^/*Hr*^*Hp *^mice

The skin was gathered from the buttocks of mice at E (Embryonic day)18.5, P0(Postnatal day 0) and P3 of wild-type (+/+) and *Hr^Hp^/Hr^Hp ^*as previously described [15]. Paraffin sections were prepared, cut 5-μm in thickness and stained with hematoxylin and eosin (H&E), following a standard method. The histological morphology was observed with an optical microscope (Olympus).

### Western blot analysis

Protein extracts were prepared from mouse skin at P0, and P3 in RIPA buffer (150 mM sodium chloride, 1% NP-40, 0.5% sodium deoxycholate, 0.1% SDS, 50 mM Tris-HCl pH8.0) following a standard method. Bradford assay was performed to quantify protein amount. Two hundred micrograms of protein were used for western blot analysis as described earlier [16]. Rabbit polyclonal HR antibody [16] and α- tubulin antibody (Santa Cruz) were used at a dilution of 1: 5000 and 1:2500, respectively. Antigen-antibody complexes were detected using ECL system (Amersham Bioscience) and exposure to x-ray film (Kodak).

### Microarray hybridization and data analysis

Total RNA was extracted from the skins of wild type and *Hr^Hp^/Hr^Hp ^*mice at P0 (N = 6) using TRIZOL following the manufacturer's instructions (Invitrogen). RNA that passed the quality check using an Agilent Bioanalyzer were used for microarray analysis using Mouse WG-6 v2.0 Expression BeadChip Kits (Illumina). Total RNA was reverse transcribed to cDNAs which were used to synthesize biotin labeled cRNA in an *in vitro *transcription reaction (Ambion). Two micrograms of biotin-labeled cRNA were loaded on to BeadChip and hybridization and washing experiments were carried out following the protocol from the manufacturer (Illumina). The slides were scanned using Illumina BeadArray Reader.

### Gene pathway analysis

To analyze biological pathways associated with differentially expressed genes in the mutant skin, we input a total of 306 genes (>1.5 fold, *p *< 0.05) to the KEGG database http://www.genome.jp/kegg/pathway.html

### Statistical analysis

The 'affy' and 'gcrma' packages of Bioconductor were used to preprocess and normalize the data following import of CEL files into the R statistical package (Affymetrix, Inc, Santa Clara, CA, USA). The GC Robust Multiarray Analysis (GC-RMA) was used to adjust perfect match (PM) probe data for background noise. Normalization was performed on adjusted perfect match (PM) data with an algorithm based on rank invariant probes [32].

After normalization, differential gene expression between groups was assessed by Significance Analysis of Microarrays (SAM) [33]. The t-test was calculated for statistical comparisons, and *p*-values were obtained with 100 permutations. The *q*-value, which is a Bayesian equivalent to the false discovery rate (FDR)-adjusted *p*-value, is estimated [34]. The *q*-value is a well suited measure of significance for the genomewide tests of significance.

### RT-PCR and Realtime PCR

Total RNA was extracted from the skins of wild type and *Hr^Hp^/Hr^Hp ^*mice at P0 (N = 6), P3 (N = 3), P7 (N = 3), P10 (N = 3) and P14 (N = 3) as described above. Single stranded cDNAs were synthesized using the PrimeScript 1^st ^strand cDNA Synthesis kit (Takara). PCR was performed using Peltier Thermal Cycler-100 (MJ Research). PCR conditions were 2 min at 95°C followed by 28 cycles of 15 sec at 94°C, 15 sec at 62°C, 15 sec at 72°C. The final extension was for 10 min at 72°C. Forward and reverse primer sequences of each gene are listed in Additional file 3. Realtime PCR was performed with SYBR Premix Ex Taq (Takara) using an Mx3000P (Stratagene). The cycling condition was initial denaturation for 2 min at 95°C followed by 45 cycles of 15 sec at 94°C, 15 sec at 62°C and 15 sec at 72°C. The gene expression levels were measured by the comparative ∆∆Ct method [35], and the relative mRNA expression levels were determined based on the realtime PCR performed in duplicate using three independent samples. Statistical significance was determined by a student t-test using Sigma plot.

### Cell culture and transient transfection experiment

Mouse keratinocyte cells (PAM212 cell line) were cultured in DMEM (Invirogen) containing 10% FBS with 5% CO_2 _at 37°C incubator. An *Hr *full-length cDNA clone (BC049182) and pcDNA 3.1(+) vector were purchased from Invitrogen. Transfection was carried out using polyethyleneimine (Sigma-Aldrich) following the manufacturer's instruction. 8 x 10^5 ^cells were seeded in 60 mm dishes in triplicate. Twenty-four hours later, 3 μg of *Hr *cDNA construct and 0.2 μg of pCMV3.1/β-gal were introduced into cells, which were harvested 48 hr post transfection and protein and total RNAs were extracted for western blot and realtime PCR analyses, respectively. Plasmid pcDNA 3.1 DNA was used as a control, and the relative expression level was normalized against transfection efficiency determined by β-Galactosidase activity.

## Authors' contributions

The research was conducted under SJKY's direction. BKK performed all experiments and analyzed data. ICB and HWL prepared skin sample and performed histological experiment. JKK and SJKY helped in drafting and revising manuscript. HSS analyzed the microarray data and calculate *p*- and *q*-values. BKK and SJKY wrote paper. All authors read and approved the final manuscript.

## Supplementary Material

Additional file 1 **Up-regulated genes in the skin of *Hr^Hp^/Hr^Hp ^*at P0 compared with that of age matched wild type (>1.2-fold, *p *and *q *< 0.05)**. click here for file

Additional file 2 **Down-regulated genes in the skin of *Hr^Hp^/Hr^Hp ^*at P0 compared with that of age matched wild type (>1.2-fold, *p *and *q *< 0.05)**. click here for file

Additional file 3 **List of gene-specific primers**. click here for file
