# Supplementary material for: Gene expression profile of the skin in the 'hairpoor' (HrHp) mice by microarray analysis
Source: BMC Genomics. 2010 Nov 18;11:640. doi: 10.1186/1471-2164-11-640 (PMC3091768; doi:10.1186/1471-2164-11-640)
Supplement: Additional file 1 — Up-regulated genes in the skin of HrHp/HrHp at P0 compared with that of age matched wild type (>1.2-fold, p and q < 0.05). [file 1471-2164-11-640-S1.DOC]

**Additional file 1** Up-regulated genes in the skin of *Hr^Hp^/Hr^Hp^* at P0 compared with that of age matched wild type (>1.2-fold, *p* and *q*<0.05)

| TargetD | Name | Fold change | p-value | q-value |
| --- | --- | --- | --- | --- |
| 1500259 | LOC382159 | 1.203518 | 1.61E-04 | 0.031621 |
| 2480059 | Anxa5 | 1.205637 | 0.003406 | 0.045386 |
| 2810142 | D330004B08Rik | 1.206153 | 4.21E-06 | 0 |
| 2760139 | A830039N02Rik | 1.206994 | 6.36E-04 | 0.034432 |
| 7210100 | 6330416L11Rik | 1.207851 | 0.003114 | 0.036594 |
| 5960221 | Slc7a2 | 1.212547 | 8.55E-04 | 0.040726 |
| 4040689 | 1700041B20Rik | 1.212663 | 4.69E-04 | 0.036594 |
| 2480682 | H60 | 1.213508 | 1.50E-05 | 0.023945 |
| 1190066 | 4930432B04Rik | 1.21379 | 0.001069 | 0.042707 |
| 1710382 | Osbpl1a | 1.215773 | 5.94E-04 | 0.036594 |
| 4780356 | 4930455F23Rik | 1.218151 | 8.99E-04 | 0.034432 |
| 2450601 | Rapgef4 | 1.218442 | 0.001142 | 0.040786 |
| 580259 | Catnd2 | 1.218937 | 0.002616 | 0.037146 |
| 2140458 | A630054M16Rik | 1.228408 | 0.001562 | 0.039648 |
| 6560605 | Mmp16 | 1.228956 | 0.00673 | 0.049529 |
| 7150397 | G431001E03Rik | 1.231426 | 2.38E-04 | 0.024642 |
| 5810593 | Traf4 | 1.23282 | 8.04E-04 | 0.034749 |
| 5960128 | LOC232875 | 1.233293 | 0.007151 | 0.0337 |
| 5870482 | Toe1 | 1.235826 | 0.002886 | 0.037215 |
| 1400168 | Bcl2 | 1.236633 | 5.27E-05 | 0.023945 |
| 2340010 | B430315A04Rik | 1.237675 | 6.35E-04 | 0.036594 |
| 4560768 | 5730466H23Rik | 1.238298 | 5.43E-04 | 0.036594 |
| 1340450 | D6Ertd245e | 1.239664 | 2.41E-04 | 0.024905 |
| 3990170 | Ebf1 | 1.23971 | 0.003386 | 0.031621 |
| 4210138 | Tes | 1.241286 | 5.36E-04 | 0.031621 |
| 4850674 | Usp39 | 1.241792 | 6.72E-04 | 0.023945 |
| 1690239 | Ptprz1 | 1.243914 | 0.002294 | 0.036594 |
| 3170280 | Col6a1 | 1.245844 | 0.002274 | 0.046255 |
| 7380670 | Asph | 1.247456 | 0.001474 | 0.036594 |
| 3890041 | Ddx24 | 1.247527 | 0.003806 | 0.036594 |
| 4880113 | 2610528C06Rik | 1.248136 | 0.004361 | 0.049952 |
| 2370484 | 9930033H14Rik | 1.24871 | 0.001488 | 0.04293 |
| 540048 | Xpnpep1 | 1.249043 | 1.28E-04 | 0.023945 |
| 2760687 | 2900024P20Rik | 1.251376 | 0.002918 | 0.046255 |
| 4640167 | D230048N11Rik | 1.2521 | 0.002147 | 0.042166 |
| 4860202 | B130020A07Rik | 1.255112 | 2.31E-04 | 0.031621 |
| 2680692 | P4hb | 1.258102 | 0.001609 | 0.0479 |
| 5560537 | 2610019N13Rik | 1.258362 | 0.001451 | 0.046765 |
| 5050228 | Pitpn | 1.260404 | 0.006455 | 0.04293 |
| 2490091 | Bbx | 1.264499 | 0.001727 | 0.039648 |
| 2570148 | Cspg2 | 1.268055 | 0.002141 | 0.046255 |
| 6510717 | Dgkh | 1.274541 | 0.001268 | 0.042166 |
| 5900376 | Stx5a | 1.274693 | 0.002852 | 0.046348 |
| 1990519 | Ntng1 | 1.277159 | 0.006073 | 0.046766 |
| 2900519 | scl0001259.1_60 | 1.279298 | 0.004293 | 0.047689 |
| 290670 | Dock10 | 1.280937 | 0.001969 | 0.046765 |
| 1850333 | Trrp1 | 1.281621 | 3.66E-04 | 0.031621 |
| 6660114 | Spo11 | 1.292343 | 2.87E-04 | 0.0337 |
| 6380470 | Itih5 | 1.297007 | 5.35E-04 | 0.036594 |
| 4810286 | Myd88 | 1.298058 | 0.002977 | 0.048711 |
| 3990431 | A630099L06Rik | 1.298696 | 0.001305 | 0.036594 |
| 4280053 | Sec61a2 | 1.299113 | 0.001924 | 0.036594 |
| 4230167 | D930044O18Rik | 1.299771 | 0.003749 | 0.049557 |
| 5050066 | Ppm1l | 1.30692 | 0.003937 | 0.031621 |
| 6400706 | 8430421H08Rik | 1.309007 | 0.00217 | 0.040833 |
| 3850440 | A930005I04Rik | 1.309013 | 0.001541 | 0.036594 |
| 3310181 | Actr1a | 1.309988 | 0.009703 | 0.046255 |
| 7570372 | 2010309L07Rik | 1.310261 | 0.001147 | 0.041305 |
| 1990021 | LOC545007 | 1.310742 | 2.63E-04 | 0.0337 |
| 5870184 | Rab6 | 1.31118 | 0.00894 | 0.046255 |
| 290300 | Sp5 | 1.792365 | 0.001775 | 0.036594 |
| 620274 | 5330439J01Rik | 1.3121 | 0.003498 | 0.046348 |
| 5340612 | D1Ertd471e | 1.318887 | 0.002923 | 0.042285 |
| 7150377 | Cd83 | 1.318943 | 9.59E-04 | 0.040726 |
| 2100253 | Zhx3 | 1.321493 | 0.001395 | 0.037146 |
| 6020431 | 4632418H02Rik | 1.322446 | 3.50E-04 | 0.023945 |
| 2350187 | Rnf4 | 1.324709 | 0.00328 | 0.048711 |
| 2360398 | scl000449.1_2 | 1.326463 | 0.002221 | 0.023945 |
| 6650039 | Raver2 | 1.329065 | 0.006348 | 0.040726 |
| 7570598 | Hey1 | 1.33095 | 3.66E-04 | 0.036594 |
| 4280494 | Slc30a5 | 1.332602 | 0.002046 | 0.046348 |
| 1410224 | EG243881 | 1.332703 | 0.0102 | 0.047689 |
| 6520603 | Reck | 1.333541 | 0.001693 | 0.046255 |
| 3460333 | Golph2 | 1.338046 | 0.005291 | 0.041236 |
| 6620114 | D830014B20Rik | 1.343039 | 0.001237 | 0.042707 |
| 3460594 | Trim33 | 1.350809 | 0.0019 | 0.048371 |
| 1660504 | Il7r | 1.353695 | 3.70E-04 | 0.034749 |
| 4890139 | Lrrc49 | 1.354171 | 0.004102 | 0.036594 |
| 7570671 | C530043G21Rik | 1.364326 | 9.23E-04 | 0.040726 |
| 5690593 | Tes | 1.366343 | 0.001982 | 0.048147 |
| 580746 | Rasgrf1 | 1.367481 | 0.004806 | 0.046255 |
| 1070646 | Ankfy1 | 1.369154 | 0.001056 | 0.031776 |
| 6590368 | Ell3 | 1.370418 | 0.002117 | 0.046766 |
| 5420605 | Tnc | 1.371414 | 3.72E-04 | 0.023945 |
| 2140102 | 0910001A06Rik | 1.374552 | 0.001239 | 0.03715 |
| 4290162 | Smad3 | 1.385849 | 0.002139 | 0.040726 |
| 6520246 | LOC98434 | 1.391231 | 0.003807 | 0.046255 |
| 580717 | Uhrf2 | 1.391646 | 9.96E-04 | 0.040726 |
| 2350646 | Akap11 | 1.392382 | 8.94E-04 | 0.03715 |
| 6900180 | Asph | 1.393446 | 0.009832 | 0.040726 |
| 6860333 | A130099L09Rik | 1.396766 | 5.09E-04 | 0.034432 |
| 6330452 | Slc35d1 | 1.39975 | 9.80E-04 | 0.03715 |
| 4610674 | Ckmt1 | 1.405074 | 8.19E-05 | 0.023945 |
| 2810603 | AW536289 | 1.409657 | 0.001623 | 0.047689 |
| 1710162 | Fer1l3 | 1.410391 | 8.65E-04 | 0.040726 |
| 840475 | Tax1bp1 | 1.412185 | 9.99E-04 | 0.040726 |
| 6560477 | Stk2 | 1.419962 | 0.00521 | 0.043696 |
| 4570278 | Krt15 | 1.421589 | 6.24E-04 | 0.037146 |
| 6480554 | BC023892 | 1.421948 | 7.08E-04 | 0.038338 |
| 6980154 | Vwa2 | 1.429539 | 8.30E-04 | 0.036594 |
| 4560465 | 8430403M15Rik | 1.435287 | 0.003255 | 0.039648 |
| 1780156 | Maged1 | 1.437094 | 0.005787 | 0.037146 |
| 1770592 | 1200013B22Rik | 1.442367 | 0.003228 | 0.041068 |
| 2570309 | Robo2 | 1.445943 | 0.001893 | 0.024905 |
| 6980612 | Lrrc1 | 1.446231 | 0.002788 | 0.036594 |
| 5560204 | 9930031P18Rik | 1.449604 | 0.001797 | 0.038338 |
| 2470753 | AI415330 | 1.449709 | 8.50E-04 | 0.036594 |
| 5220475 | B230373P09Rik | 1.4583 | 0.001842 | 0.037146 |
| 3400487 | Thsd2 | 1.458432 | 0.003226 | 0.048147 |
| 5720619 | 4732465J09Rik | 1.463987 | 0.001061 | 0.040726 |
| 840243 | Hectd2 | 1.46851 | 0.004611 | 0.036594 |
| 2320273 | Dock11 | 1.469417 | 0.00593 | 0.04293 |
| 1740541 | Adcy2 | 1.475829 | 6.70E-04 | 0.03715 |
| 6770139 | Hrb | 1.47794 | 6.64E-04 | 0.0337 |
| 6520154 | BC027231 | 1.480934 | 9.54E-04 | 0.034432 |
| 2630040 | A130026C10Rik | 1.481753 | 0.001287 | 0.037215 |
| 2690689 | Elys | 1.48912 | 0.016317 | 0.047689 |
| 2480129 | Pmp22 | 1.492014 | 0.001355 | 0.040786 |
| 5570240 | BB128963 | 1.494299 | 0.002631 | 0.046255 |
| 870309 | Serpine2 | 1.495062 | 3.44E-04 | 0.031621 |
| 3170465 | AI505012 | 1.49809 | 0.00212 | 0.035821 |
| 3800228 | Tmem23 | 1.501213 | 7.39E-04 | 0.036594 |
| 510041 | Epb4.1l3 | 1.502335 | 0.011583 | 0.046765 |
| 4640376 | Zdhhc17 | 1.502478 | 0.004006 | 0.041305 |
| 5310050 | Jak2 | 1.514057 | 0.001914 | 0.040726 |
| 4010056 | Pdgfc | 1.516232 | 4.78E-05 | 0.023945 |
| 2120634 | Serpine2 | 1.529581 | 3.21E-04 | 0.034981 |
| 2060592 | Ddah1 | 1.530376 | 5.01E-05 | 0.023945 |
| 5490347 | Sestd1 | 1.530957 | 1.53E-04 | 0.023945 |
| 5890273 | Has3 | 1.531682 | 0.002551 | 0.046395 |
| 3310441 | Twsg1 | 1.532081 | 0.008884 | 0.045753 |
| 1030598 | D630029N22Rik | 1.542926 | 5.76E-04 | 0.036594 |
| 4610095 | Dnajc3 | 1.545027 | 0.002355 | 0.049529 |
| 7510154 | Ets2 | 1.54861 | 0.001106 | 0.040726 |
| 6060452 | Fgfr2 | 1.551387 | 0.002096 | 0.047689 |
| 2760431 | Jak2 | 1.565484 | 0.004102 | 0.047689 |
| 6380632 | Cspg2 | 1.583869 | 0.003439 | 0.040726 |
| 510240 | 4732455L03Rik | 1.602204 | 0.005878 | 0.040833 |
| 5910241 | Peg3 | 1.605959 | 0.011291 | 0.046765 |
| 1440286 | Hectd2 | 1.606717 | 0.001983 | 0.036594 |
| 4860441 | Rftn2 | 1.608498 | 0.006128 | 0.036594 |
| 3290133 | Enpp1 | 1.615265 | 0.001235 | 0.037611 |
| 3060670 | 9430089E08Rik | 1.625655 | 0.009835 | 0.046766 |
| 6130433 | Eppk1 | 1.646172 | 0.00161 | 0.042166 |
| 1170626 | Pik3r1 | 1.652784 | 0.001358 | 0.043209 |
| 4640397 | Nin | 1.654658 | 0.003007 | 0.04293 |
| 110347 | Pcdh20 | 1.671517 | 8.46E-05 | 0.023945 |
| 510504 | 3110068G20Rik | 1.693148 | 0.002085 | 0.039648 |
| 5810039 | Lama3 | 1.697899 | 0.002047 | 0.039648 |
| 3870670 | Thbs1 | 1.704254 | 8.69E-04 | 0.038338 |
| 360445 | Cd109 | 1.710668 | 7.10E-04 | 0.031776 |
| 6270682 | Slc15a2 | 1.762516 | 1.53E-04 | 0.031621 |
| 6660292 | Spo11 | 1.780484 | 0.002529 | 0.034432 |
| 4290300 | Sp5 | 1.792365 | 0.002255 | 0.038338 |
| 3140370 | Nid1 | 1.820393 | 0.002088 | 0.04293 |
| 1850687 | Mef2a | 1.822892 | 0.001537 | 0.040726 |
| 1850215 | Zfp288 | 1.82622 | 4.35E-04 | 0.034981 |
| 160066 | Smpd3 | 1.841468 | 1.70E-04 | 0.023945 |
| 430008 | Lrrn1 | 2.066969 | 0.00495 | 0.03715 |
| 6840121 | Odc1 | 2.102129 | 2.28E-04 | 0.021604 |
| 6280392 | Hmgcs2 | 2.166179 | 1.57E-04 | 0.023945 |
| 6290592 | LOC226691 | 2.219279 | 7.06E-04 | 0.036594 |
| 1450491 | Serpina3h | 2.963004 | 0.00377 | 0.039382 |
