# Supplementary material for: Gene expression profile of the skin in the 'hairpoor' (HrHp) mice by microarray analysis
Source: BMC Genomics. 2010 Nov 18;11:640. doi: 10.1186/1471-2164-11-640 (PMC3091768; doi:10.1186/1471-2164-11-640)
Supplement: Additional file 2 — Down-regulated genes in the skin of HrHp/HrHp at P0 compared with that of age matched wild type (>1.2-fold, p and q < 0.05). [file 1471-2164-11-640-S2.DOC]

**Additional file 2** Down-regulated genes in the skin of *Hr^Hp^/Hr^Hp^* at P0 compared with that of age matched wild type (>1.2-fold, *p* and *q*<0.05)

| TargetD | Name | Fold change | p-value | q-value |
| --- | --- | --- | --- | --- |
| 3780021 | Cidea | 0.147951 | 0.003652 | 0.042166 |
| 2710593 | Cyp2g1 | 0.177938 | 1.29E-04 | 0.023945 |
| 2000592 | Cyp2g1 | 0.209315 | 2.13E-04 | 0.023945 |
| 7570053 | BC018222 | 0.243462 | 4.54E-04 | 0.031621 |
| 2260040 | Foxe1 | 0.276223 | 6.41E-05 | 0.012603 |
| 3060095 | Krt71 | 0.305014 | 0.00197 | 0.048661 |
| 5080575 | Sct | 0.351445 | 1.42E-04 | 0.031326 |
| 7380014 | LOC384538 | 0.364737 | 0.002539 | 0.04293 |
| 5090202 | LOC381019 | 0.382106 | 6.05E-04 | 0.023945 |
| 2810706 | Fa2h | 0.38835 | 0.002569 | 0.04293 |
| 2070669 | Klk6 | 0.396191 | 4.11E-04 | 0.034432 |
| 6560093 | Sprr1a | 0.406979 | 1.72E-04 | 0.031621 |
| 1690201 | Padi1 | 0.418053 | 2.69E-04 | 0.031621 |
| 940338 | S100a1 | 0.433973 | 7.18E-04 | 0.038739 |
| 6770347 | LOC208963 | 0.434611 | 0.001511 | 0.038338 |
| 6900440 | Eraf | 0.442446 | 4.23E-04 | 0.036594 |
| 1990373 | Areg | 0.470272 | 8.89E-05 | 0.023945 |
| 6290411 | Pank1 | 0.48369 | 0.001969 | 0.047745 |
| 7330112 | Anxa5 | 0.50062 | 0.003076 | 0.036594 |
| 5130372 | LOC224046 | 0.504916 | 0.003454 | 0.03715 |
| 2030370 | BC039632 | 0.521599 | 0.001333 | 0.036594 |
| 5420347 | Vdr | 0.536069 | 0.002599 | 0.0479 |
| 3440132 | Smpdl3b | 0.537679 | 8.20E-04 | 0.036594 |
| 4260195 | Fblim1 | 0.546164 | 0.003933 | 0.036594 |
| 3890309 | 1700081D17Rik | 0.547143 | 1.06E-04 | 0.024905 |
| 1260113 | 9930017A07Rik | 0.554767 | 2.97E-04 | 0.034749 |
| 1820592 | 2210008A03Rik | 0.555196 | 1.49E-04 | 0.030606 |
| 2140215 | 2010004M13Rik | 0.573242 | 9.99E-04 | 0.040726 |
| 2510037 | Alad | 0.574142 | 0.002354 | 0.046596 |
| 1690600 | AI646023 | 0.574559 | 0.002464 | 0.040726 |
| 6550164 | E2f2 | 0.590729 | 6.73E-04 | 0.0337 |
| 770162 | E2f2 | 0.598475 | 0.002121 | 0.04293 |
| 3830427 | Cmtm8 | 0.618379 | 0.006232 | 0.045386 |
| 2100162 | Dhcr24 | 0.623409 | 1.40E-04 | 0.031621 |
| 670653 | 1810021J13Rik | 0.818189 | 2.58E-04 | 0.0337 |
| 1740475 | 5430426F23Rik | 0.638704 | 0.002572 | 0.041236 |
| 1570220 | Hoxb6 | 0.643198 | 0.001002 | 0.036594 |
| 1070670 | 2210415K03Rik | 0.646253 | 0.001625 | 0.036594 |
| 2750184 | 1810054O13Rik | 0.646966 | 6.76E-04 | 0.027005 |
| 2810494 | E2f2 | 0.648629 | 1.01E-04 | 0.024905 |
| 2750753 | Slc25a39 | 0.649764 | 0.002712 | 0.040726 |
| 840673 | Lmnb1 | 0.651103 | 0.006905 | 0.038338 |
| 7320753 | Hsd17b2 | 0.658519 | 5.35E-04 | 0.036594 |
| 3370762 | 4632428M11Rik | 0.6608 | 1.17E-04 | 0.023945 |
| 650324 | Prss22 | 0.661676 | 8.37E-05 | 0.023945 |
| 4730520 | Cox5a | 0.66488 | 4.55E-04 | 0.036594 |
| 360025 | Hoxb2 | 0.667371 | 0.001076 | 0.0337 |
| 4880326 | Them2 | 0.668778 | 0.002195 | 0.048795 |
| 4220056 | Tpm1 | 0.67024 | 7.35E-04 | 0.034432 |
| 5290711 | Car2 | 0.671227 | 7.67E-04 | 0.039648 |
| 6350114 | Ube2l6 | 0.674026 | 0.001557 | 0.047689 |
| 7330070 | Cmtm8 | 0.675981 | 0.003752 | 0.046348 |
| 3370243 | Smtn | 0.67819 | 0.001175 | 0.040726 |
| 5690408 | Exosc5 | 0.685912 | 0.007431 | 0.047858 |
| 6370204 | Tagln2 | 0.686822 | 0.001446 | 0.036594 |
| 940692 | Cox7b | 0.696772 | 0.001782 | 0.047689 |
| 1050538 | BC009118 | 0.698576 | 0.003136 | 0.037146 |
| 7210669 | Extl1 | 0.700305 | 0.002556 | 0.03715 |
| 4590010 | 2410015C20Rik | 0.700445 | 2.06E-04 | 0.031776 |
| 3420564 | Sgk3 | 0.701228 | 0.001314 | 0.046255 |
| 1440040 | Garnl4 | 0.708858 | 5.69E-04 | 0.03703 |
| 4060398 | Sfrp1 | 0.710293 | 0.002421 | 0.023945 |
| 4060484 | Tpm1 | 0.711281 | 8.66E-04 | 0.03715 |
| 4120692 | Fahd1 | 0.712423 | 0.010752 | 0.04293 |
| 5700494 | Fut4 | 0.713273 | 2.72E-04 | 0.023945 |
| 780195 | Tmem53 | 0.713975 | 4.97E-04 | 0.036594 |
| 130609 | BC026432 | 0.714421 | 7.72E-04 | 0.037215 |
| 3870681 | Slc7a8 | 0.715095 | 9.73E-04 | 0.037215 |
| 460692 | Bcl11a | 0.717322 | 0.00184 | 0.0479 |
| 3310025 | D4Bwg0951e | 0.723247 | 0.003333 | 0.040786 |
| 4010241 | LOC384801 | 0.728793 | 0.001155 | 0.04293 |
| 3060209 | mt-Nd4l | 0.731442 | 7.40E-04 | 0.034432 |
| 6130411 | Tagln | 0.732959 | 0.001007 | 0.041305 |
| 3420544 | Tmepai | 0.734024 | 0.001119 | 0.037215 |
| 2490019 | Gstt2 | 0.735596 | 9.46E-04 | 0.040726 |
| 5260204 | Pdlim2 | 0.740581 | 2.15E-04 | 0.031776 |
| 2600274 | LOC233080 | 0.747245 | 2.18E-04 | 0.023945 |
| 160370 | Mrpl3 | 0.749195 | 0.002331 | 0.04293 |
| 3130437 | Hras1 | 0.751104 | 0.002421 | 0.046255 |
| 3310577 | Gna15 | 0.752648 | 5.66E-04 | 0.036594 |
| 5570767 | Nudt14 | 0.758098 | 0.001344 | 0.039648 |
| 60192 | Cbx8 | 0.758458 | 2.76E-04 | 0.0337 |
| 2570139 | Itgb6 | 0.760475 | 0.0034 | 0.047447 |
| 5870470 | Arhgap9 | 0.761045 | 3.15E-05 | 0.023945 |
| 3990270 | Rad1 | 0.76205 | 2.85E-04 | 0.031621 |
| 430307 | Zfp219 | 0.762145 | 0.002621 | 0.040726 |
| 6040286 | B230312A22Rik | 0.764142 | 0.001279 | 0.042166 |
| 4670047 | 1810009H17Rik | 0.766968 | 0.002706 | 0.047537 |
| 6580341 | Cyp11a1 | 0.767346 | 3.78E-04 | 0.024642 |
| 840477 | scl0015365.1_6 | 0.768049 | 0.0033 | 0.045386 |
| 5810044 | Rarg | 0.768107 | 0.001454 | 0.037215 |
| 6020187 | Piwil2 | 0.769963 | 0.001265 | 0.036594 |
| 7210497 | BC003251 | 0.771848 | 0.002955 | 0.036594 |
| 670242 | Gpaa1 | 0.773044 | 0.002606 | 0.046255 |
| 2480088 | Sertad3 | 0.774516 | 0.003237 | 0.039992 |
| 2970091 | 1810021J13Rik | 0.775013 | 2.92E-04 | 0.034432 |
| 5090538 | Prss25 | 0.780699 | 4.47E-04 | 0.036594 |
| 5090670 | Rhov | 0.782004 | 0.002391 | 0.040726 |
| 4560220 | Ppa2 | 0.784388 | 4.11E-04 | 0.036594 |
| 6860408 | Zfp187 | 0.785861 | 4.33E-04 | 0.021604 |
| 3610634 | C130020C07Rik | 0.786854 | 8.28E-04 | 0.036594 |
| 5090181 | Trrp2 | 0.791606 | 0.001445 | 0.046255 |
| 6480288 | Aldh4a1 | 0.792013 | 0.001539 | 0.047689 |
| 1780047 | LOC244710 | 0.792401 | 8.72E-04 | 0.037146 |
| 6450278 | Lgals4 | 0.794326 | 0.001407 | 0.037215 |
| 2710451 | LOC210582 | 0.800626 | 0.003022 | 0.047689 |
| 2600360 | Matn4 | 0.804313 | 0.002922 | 0.047537 |
| 5870722 | Sec14l1 | 0.804425 | 0.010953 | 0.047858 |
| 60041 | Il11ra1 | 0.807672 | 4.43E-04 | 0.036594 |
| 1070070 | Gtf3c1 | 0.808372 | 2.87E-04 | 0.0337 |
| 1070243 | Depdc6 | 0.809207 | 0.002267 | 0.040726 |
| 2450768 | Zfp213 | 0.809458 | 0.00126 | 0.0337 |
| 6330730 | Ndufa9 | 0.809768 | 0.001813 | 0.041305 |
| 1940228 | Ankrd54 | 0.815561 | 0.003653 | 0.046255 |
| 1570202 | Cmas | 0.818327 | 5.26E-04 | 0.036594 |
| 6480575 | Slc12a9 | 0.818824 | 6.62E-04 | 0.036594 |
| 1110615 | Abcb9 | 0.819221 | 0.002423 | 0.049557 |
| 7200243 | Acot8 | 0.822514 | 0.002805 | 0.040726 |
