# Supplementary material for: Gene expression profile of the skin in the 'hairpoor' (HrHp) mice by microarray analysis
Source: BMC Genomics. 2010 Nov 18;11:640. doi: 10.1186/1471-2164-11-640 (PMC3091768; doi:10.1186/1471-2164-11-640)
Supplement: Additional file 3 — List of gene-specific primers. [file 1471-2164-11-640-S3.DOC]

**Additional file 3** List of gene-specific primers

| **Genes** | **Sequences** | **Genbank accession Number** |
| --- | --- | --- |

| *Wif1* | F:caacaagtgccagtgtcgag | NM_011915 |
| --- | --- | --- |
|  | R:gcatttgaacatccaacacg | |
| *Sfrp1* | F:tcagaggccatcattgaaca | NM_013834 |
|  | R:gcaggtactggctcttcacc | |
| *Casp14* | F:gatgaggttgctgtgctcaa | NM_009809 |
|  | R:cctccgtgtttgccataagt | |
| *Fgf10* | F:gtcaaagccatcaacagcaa | NM_008002 |
|  | R: ccattgtgctgccagttaaa | |
| *Krt15* | F:gaacaaggaggtggcgtcta | NM_008469 |
|  | R:ggtaatgaccccctggatct | |
| *Wnt7b* | F:tccgagtagggagtcgagag | NM_009258 |
|  | R:agaaaagtcgatgccgtagc | |
| *Jak2* | F:atctggtacccacccaatca | NM_008413 |
|  | R:tccatccgtgaacaaaatca | |
| *Krt71* | F:tcagatccagtcccacatca | NM_019956 |
|  | R:gtacagggcctcagcttcag | |
| *Gapdh* | F:aactttggcattgtggaagg | NM_008084 |
|  | R:acacattgggggtaggaaca | |
